# Circulating TNF receptor levels are associated with estimated glomerular filtration rate even in healthy individuals with normal kidney function

**DOI:** 10.1038/s41598-024-57265-x

**Published:** 2024-03-27

**Authors:** Tomohito Gohda, Maki Murakoshi, Terumi Shibata, Yusuke Suzuki, Hiroyuki Takemura, Koji Tsuchiya, Tomoki Okada, Mitsuru Wakita, Yuki Horiuchi, Yoko Tabe, Nozomu Kamei

**Affiliations:** 1 Department of Nephrology, Juntendo University Faculty of Medicine, Hongo 2-1-1, Bunkyo-Ku, Tokyo, 113-8421 Japan; 2 Department of Clinical Laboratory, Juntendo University Hospital, Bunkyo-Ku, Tokyo, Japan; 3 Department of Clinical Laboratory Medicine, Juntendo University Faculty of Medicine, Bunkyo-Ku, Tokyo, Japan; 4 Department of Endocrinology and Metabolism, Hiroshima Red Cross Hospital & Atomic-Bomb Survivors Hospital, Hiroshima, Japan; 5 Institute for Clinical Research, National Hospital Organization, Kure Medical Center and Chugoku Cancer Center, Hiroshima, Japan

**Keywords:** TNF receptor, Biomarker, Diabetes, eGFR, Kidney function, Healthy subject, Biomarkers, Endocrinology, Nephrology

## Abstract

The association between serum tumor necrosis factor receptor (TNFRs: TNFR1, TNFR2) levels and estimated glomerular filtration rate (eGFR) observed in patients with diabetes has not been comprehensively tested in healthy subjects with normal kidney function. It also remains unclear whether TNFR levels differ by age and sex, and between healthy subjects and diabetics. We measured serum TNFR levels in 413 healthy subjects and 292 patients with type 2 diabetes. In healthy subjects, TNFR levels did not differ between men and women. Additionally, TNFR2, but not TNFR1, levels increased with age. In multivariate analysis, TNFR1 was associated only with cystatin C-based eGFR (eGFR-CysC), whereas TNFR2 was associated with systolic blood pressure in addition to eGFR-CysC. Both TNFRs were associated with lower eGFR (eGFR-Cys < 90 mL/min/1.73 m^2^) even after adjustment for relevant clinical factors. Upon combining healthy subjects and patients with diabetes, the presence of diabetes and elevated glycated hemoglobin level were significant factors in determining TNFR levels. TNFR levels were associated with eGFR-CysC, but were not affected by age and sex in healthy subjects with normal kidney function. TNFR levels in patients with diabetes appeared to be higher than in healthy subjects.

## Introduction

A growing body of evidence suggests that chronic inflammation plays an important role in the progression of chronic kidney disease (CKD), including diabetic kidney disease (DKD)^1,2^. Tumor necrosis factor (TNF)α is a central proinflammatory cytokine, which binds functionally distinct cell surface receptors, TNF receptors (TNFRs: TNFR1 and TNFR2)^3,4^. A number of studies have reported that elevated levels of TNFR predict decline in kidney function in patients with diabetes and CKD, and even in the general population^5–11^. TNFR levels correlate with both albuminuria/proteinuria and estimated glomerular filtration rate (eGFR)^12,13^. Moreover, several studies have reported that other factors, such as age, sex, body mass index (BMI), hypertension, glycated hemoglobin (HbA1c), high-density-lipoprotein cholesterol (HDL-C), and uric acid, also correlate with TNFR levels in patients with diabetes^14,15^.

However, it has not been comprehensively tested whether TNFR levels are associated with urinary albumin-to-creatinine ratio (UACR) and eGFR even in healthy subjects with normal kidney function. Additionally, it is unclear whether the TNFR levels in healthy subjects differ from those in patients with diabetes. Against this background, this study was established to shed light on these issues by measuring serum TNFRs and eGFR in 413 healthy subjects and 292 patients with type 2 diabetes.

## Materials and methods

### Healthy subjects and patients with type 2 diabetes

Japanese adults who worked at Juntendo University Hospital in Tokyo, Japan, participated in this study as healthy controls. An annual health check-up is held for the staff at this hospital. We included 3861 of these individuals who expressed a willingness to participate in the study as healthy controls. Of these, we randomly recruited 910 individuals for whom UACR had been measured for each age group and sex. Individuals with a past medical history including cancer, diabetes, hypertension, kidney disease, dyslipidemia, or other major pathological conditions, or HbA1c > 6.5%, systolic blood pressure (BP) > 140 mmHg, diastolic BP > 90 mmHg, and eGFR-creatinine (Cr) < 60 mL/min/1.73 m^2^, were excluded from this study. To apply these exclusion criteria, we used data from the annual health check-up in 2022. After the application of all exclusion criteria, the final group of healthy controls was made up of 413 Japanese adults aged between 25 and 69 years (Supplementary Fig. 1). This study was approved by the institutional review board (IRB) of Juntendo University Hospital, Tokyo, Japan (No. M20-0074). The study complied with the guidelines of the Declaration of Helsinki., with subjects being able to opt out via the hospital’s website.

A separate cohort of patients with type 2 diabetes were recruited from Kure Medical Center and Chugoku Cancer Center, Hiroshima, Japan. In brief, 738 Japanese patients with diabetes agreed to participate in an observational follow-up study. Of these, we selected only 292 patients aged between 25 and 69 years, with type 2 diabetes, and eGFR-Cr > 60 mL/min/1.73 m^2^ (Supplementary Fig. 1). All patients with diabetes were undergoing treatment from a diabetes specialist, and 25.3% of them were undergoing insulin therapy. This study was approved by the ethics committee of Kure Medical Center and Chugoku Cancer Center (No. 26-06). Informed consent was obtained from all patients, and the study complied with the tenet of the Declaration of Helsinki.

### Assessment of clinical variables

Serum Cr and cystatin C (CysC) levels were measured using standard enzymatic methods, and colloidal gold immunoassay [Nescauto GC Cystatin C (Nm); Alfresa Pharma Corp., Osaka, Japan], respectively. eGFR was calculated in accordance with Japanese Society of Nephrology guidelines using the following formula, which was designed for the Japanese population: eGFR-Cr (mL/min/1.73 m^2^) = 194 × [age (years)]^−0.287^ × [serum Cr (mg/ dL)]^−1.094^ × 0.739 (for women); eGFR-CysC (mL/min/1.73 m^2^) = [104 × (serum CysC)^−1.019^ × 0.996^Age^ (× 0.929 for women)] − 8^16,17^. Urinary albumin was analyzed by a nephelometry assay (N-assay TIA Micro Alb; Nittobo Medical Co., Ltd., Fukushima, Japan). UACR was used as an index of urinary albumin excretion, and expressed as milligrams of albumin per gram of Cr (mg/gCr). Non-HDL-C was defined as the difference between total cholesterol and HDL-C.

### Measurement of tumor necrosis factor receptors 1 and 2 in serum

We measured serum TNFR1 and TNFR2 concentrations using Human TNF RI/TNFRSF1A and TNF RII/TNFRSF1B Quantikine ELISA kits (Cat. Nos. DRT100 and DRT200; R&D Systems, Minneapolis, MN, USA), respectively, as previously described^18^.

### Statistical analysis

The characteristics of the participants are expressed as mean ± standard deviation (SD), median (25–75% interquartile range), or frequency (count and percentage). We used the Shapiro–Wilk test to assess the normality of distributions. TNFR and UACR are expressed as the median and 25–75% interquartile range, and other characteristics are expressed as the mean ± SD. Continuous variables were compared using the Mann–Whitney U test. Fisher’s exact test was used to compare categorical variables. Data with two independent variables were analyzed by two-way ANOVA. Spearman’s rank correlation analysis was used to analyze the correlations between two variables. Univariate and multivariate linear regression models were used to determine the factors that affect the serum TNFR levels. Univariate and multivariate logistic regression analyses were performed to examine the association of lower eGFR-CysC and TNFR levels. Statistical analyses were performed using SAS version 9.4 (SAS Institute, Cary, NC, USA). A p value of < 0.05 was considered statistically significant.

## Results

### Clinical characteristics of healthy control and patients with diabetes

As shown in Table 1, the healthy subjects and the diabetes cohort consisted of 413 individuals (206 women, 49.9%) and 292 patients (143 women, 49.0%), respectively. We recruited healthy subjects and patients with diabetes whose eGFR-Cr was > 60 mL/min/1.73 m^2^. No significant difference was observed in the sex distribution or eGFR-Cr between the two cohorts. However, the patients with diabetes were older and had higher BMI, systolic BP, diastolic BP, HbA1c, uric acid, UACR, TNFR1, and TNFR2 than the healthy subjects. Meanwhile, healthy subjects had higher HDL-C and non-HDL-C than patients with diabetes.

**Table 1:** Clinical characteristics of healthy subjects and patients with type 2 diabetes.

Characteristics	Healthy subjects (n = 413)	Diabetics (n = 292)	U value	P value
Age (year)	43 ± 12	57 ± 11	22,292	< 0.0001
Male (%)	206 (49.9)	143 (49.0)	NA	0.81
BMI	22.1 ± 3.3	26.1 ± 5.1	29,797	< 0.0001
Systolic BP (mmHg)	120 ± 15	138 ± 16	23,709	< 0.0001
Diastolic BP (mmHg)	72 ± 11	81 ± 11	32,172	< 0.0001
HbA1c (%)	5.5 ± 0.3	7.5 ± 1.2	2365	< 0.0001
HDL-C (mg/dL)	67 ± 17	52 ± 12	92,155	< 0.0001
Non-HDL-C (mg/dL)	141 ± 35	134 ± 33	67,391	0.008
Uric acid (mg/dL)	4.9 ± 1.3	5.1 ± 1.2	53,489	0.011
UACR (mg/gCr)	4.5 (3.4, 7.3)	18.1 (7.1, 58.9)	92,155	< 0.0001
eGFR-Cr (mL/min/1.73 m^2^)	82 ± 13	85 ± 19	58,505	0.50
eGFR-CysC (mL/min/1.73 m^2^)	110 ± 19	97 ± 23	81,948	< 0.0001
TNFR1 (pg/mL)	992 (873, 1126)	1357 (1133, 1646)	19,703	< 0.0001
TNFR2 (pg/mL)	1951 (1717, 2218)	2904 (2505, 3391)	14,127	< 0.0001

*BMI* body mass index, *BP* blood pressure, *Cr* creatinine, *CysC* cystatin C, *eGFR* estimated glomerular filtration rate, *HbA1c* glycated hemoglobin, *HDL-C* high-density-lipoprotein cholesterol, *NA* not applicable, *TNF* tumor necrosis factor, *TNFR* TNF receptor, *UACR* urinary albumin-to-creatinine ratio.

### Circulating TNFR levels in each age group and sex in healthy subjects

As shown in Table 2, neither of the TNFR levels differed between the male and female groups. However, there was a significant difference in the TNFR2 levels between the age groups (p = 0.047).

**Table 2:** Clinical characteristics of healthy subjects according to sex and age group.

Variables	Women					Men					Group contrast	
	25–29 years	30–39 years	40–49 years	50–59 years	60–69 years	25–29 years	30–39 years	40–49 years	50–59 years	60–69 years	Age	Sex
Number	31	60	56	35	25	30	60	56	33	27		
TNFR1	981 (845, 1110)	998 (888, 1157)	963 (827, 1111)	1036 (906, 1147)	951 (905, 1103)	978 (884, 1143)	984 (869, 1097)	954 (861, 1107)	1023 (909, 1191)	993 (854, 1205)	0.40	0.43
TNFR2	1944 (1773, 2122)	1858 (1689, 2100)	1896 (1724, 2169)	1973 (1751, 2541)	2091 (1867, 2421)	1912 (1679, 2298)	1840 (1605, 2113)	1961 (1730, 2240)	2089 (1905, 2321)	2028 (1759, 2251)	0.047	0.63

Abbreviations used in this table are the same as in Table 1.

### Correlations among TNFRs, eGFR, and clinical parameters

**Figure 1:**
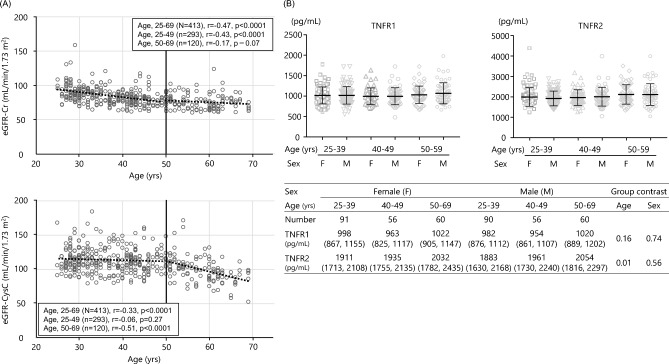
Associations of age and sex with eGFR and serum TNFR levels in healthy subjects. (**A**) Scatter plot of association between age and eGFR-Cr or eGFR-cysC. (**B**) Serum TNFR levels by each age and sex. The bars represent median and 25–75% interquartile range.

As shown in Table 3, in healthy subjects, a moderate correlation was observed between TNFR1 and TNFR2 (r = 0.58, p < 0.0001). Meanwhile, negative correlations were observed between the TNFRs and eGFR-CysC (TNFR1, r = − 0.38, p < 0.0001; TNFR2, r = − 0.35, p < 0.0001), while there were very weak correlations between the TNFRs and eGFR-Cr (TNFR1, r = − 0.10, p < 0.05; TNFR2, r = − 0.10, p < 0.05). TNFR2 was also associated with age (r = 0.11, p < 0.05) and systolic BP (r = 0.11, p < 0.05), but the correlations were very weak. The association between TNFR2 and age may have been due in part to the fact that eGFR-CysC was more strongly correlated with age for subjects over 50 years of age than for those under 50 years of age (Fig. 1). Indeed, as shown in Supplementary Table 1, multivariate regression analysis demonstrated that eGFR-CysC, but not eGFR-Cr, was associated with both TNFR1 and TNFR2. Systolic BP was still weakly associated with TNFR2, but age was not after adjustment. In patients with type 2 diabetes, TNFRs were associated with more clinical factors than in healthy subjects, in both univariate and multivariate regression analyses (Table 3 and Supplementary Table 2).

**Table 3:** Spearman's correlation between TNFRs, eGFR, and clinical parameters in healthy subjects and patients with type 2 diabetes.

Variables	Healthy subjects (n = 413)				Diabetics (n = 292)			
	TNFR1	TNFR2	GFR-Cr	GFR-CysC	TNFR1	TNFR2	GFR-Cr	GFR-CysC
Age	0.04	0.11*	− 0.47***	− 0.33***	0.14*	0.16**	− 0.48***	− 0.31***
BMI	0.08	0.05	− 0.21***	− 0.04	0.17**	0.11	0.20***	− 0.05
Systolic BP	0.07	0.11*	− 0.14**	− 0.09	0.11	0.07	− 0.07	− 0.15*
Diastolic BP	0.03	0.08	− 0.16**	− 0.07	0.05	0.04	0.09	0.09
HbA1c	0.06	0.03	− 0.18***	− 0.12*	− 0.20***	− 0.25***	0.17**	0.08
HDL-C	− 0.04	0.03	− 0.01	− 0.02	− 0.08	− 0.13*	− 0.10	− 0.19**
Non-HDL-C	0.04	0.05	− 0.23***	− 0.17***	0.19**	0.13*	0.10	0.05
Uric acid	0.01	− 0.01	− 0.12*	0.13**	0.17**	0.14*	− 0.17**	0.05
UACR	− 0.004	0.02	0.11*	− 0.20***	0.38***	0.25***	0.13*	− 0.10
eGFR-Cr	− 0.10*	− 0.10*	–	–	− 0.18**	− 0.19**	–	–
eGFR-CysC	− 0.38***	− 0.35***	0.19***	–	− 0.30***	− 0.28***	0.31***	–
TNFR2	0.58***	–	–	–	0.82***	–	–	–

Abbreviations used in this table are the same as in Table 1.

*p < 0.05, **p < 0.01, ***p < 0.001

### TNFRs as early markers of decline in kidney function

In healthy subjects, both eGFR-Cr and eGFR-CysC were associated with many demographic/clinical parameters, including age, HbA1c, non-HDL-C, uric acid, UACR, and TNFRs, although eGFR-Cr and eGFR-CysC were associated weakly with each other (r = 0.19, p < 0.001) (Table 3). Age and UACR remained significant clinical predictors of lower eGFR-CysC (< 90 mL/min/1.73 m^2^) in the multivariate analysis (model 1) (Table 4). Next, we assessed the independent effect of each TNFR on lower eGFR-CysC by adding them to model 1 (models 2–3) (Table 4). In these models, both TNFRs remained significant, in addition to age and UACR.

**Table 4:** Multivariate analysis of variables associated with eGFR decline (eGFR-CysC < 90 mL/min/1.73 m^2^) in healthy subjects.

Variables	Per 1 increase	Model 1		Model 2		Model 3	
		OR (95% CI)	P-value	OR (95% CI)	P-value	OR (95% CI)	P-value
Age	1 year	1.07 (1.03–1.10)	< 0.0001	1.08 (1.05–1.11)	< 0.0001	1.08 (1.05–1.11)	< 0.0001
Uric acid	1 mg/dL	0.79 (0.61–1.03)	0.08	–	–	–	–
HbA1c	1%	2.93 (0.85–10.09)	0.09	–	–	–	–
Non-HDL-C	1 mg/dL	1.00 (0.93–1.01)	0.57	–	–	–	–
UACR	log 1SD = 0.28	1.43 (1.09–1.89)	0.011	1.57 (1.17–2.11)	0.003	1.49 (1.12–1.99)	0.006
TNFR1	log 1SD = 0.09	–	–	2.51 (1.76–3.59)	< 0.0001	–	–
TNFR2	log1 SD = 0.09	–	–	–	–	1.88 (1.38–2.57)	< 0.0001

Model 1: age, uric acid, HbA1c, non-HDL-C, and UACR.

Model 2: age, UACR, and TNFR1.

Model 3: age, UACR, and TNFR2.

Abbreviations used in this table are the same as in Table 1.

*CI* confidence interval, *OR* odds ratio.

### TNFR levels are associated with presence of diabetes and higher glycated hemoglobin

To determine whether TNFR values are higher in patients with type 2 diabetes mellitus than in healthy controls, the two groups were combined into a single group and subjected to univariate and multivariate stepwise regression analyses to identify factors that define TNFR values. TNFRs were found to be associated with many clinical parameters (Table 5, Supplementary Tables 3 and 4). The presence of diabetes and elevated HbA1c level were revealed to be significant factors in determining TNFR levels in multivariate regression analysis, indicating that TNFR levels in patients with diabetes appeared to be higher than in healthy subjects.

**Table 5:** Multivariate stepwise regression analysis of the factors associated with tumor necrosis factor receptor 1 or tumor necrosis factor receptor 2 in the combined group of healthy subjects and patients with type 2 diabetes.

Variables	Estimate	SE	t value	P
**TNFR1**				
Age	− 0.0006	0.0340	− 1.943	0.052
Sex	0.0067	0.0070	9.556	< 0.0001
BMI	0.0016	0.0008	1.967	0.0049
Diabetes	0.0767	0.0094	8.180	< 0.0001
Non-HDL	− 0.0003	0.0001	− 2.773	0.006
eGFR-CysC	− 0.0024	0.0002	− 13.673	< 0.0001
ACR	0.0452	0.0066	6.855	< 0.0001
**TNFR2**				
Sex	0.0628	0.0074	8.526	< 0.0001
Diabetes	0.1167	0.0086	13.521	< 0.0001
Non-HDL	− 0.0003	0.0001	− 3.061	0.003
eGFR-CysC	− 0.0023	0.0002	− 12.725	< 0.0001
UACR	0.0375	0.0070	5.352	< 0.0001

Abbreviations used in this table are the same as in Table 1.

*SE* standard error.

## Discussion

In this study, we demonstrated that serum TNFR levels are associated with eGFR-CysC, but not eGFR-Cr, even in healthy subjects with normal kidney function, as well as in patients with type 2 diabetes and normal kidney function. The findings also showed that neither of the TNFRs was affected by age and sex in healthy subjects. Moreover, patients with type 2 diabetes had higher TNFR levels than healthy subjects.

Because the decline in eGFR with age is expected to affect TNFR excretion, resulting in lower fractional TNFR excretion with age, the population with eGFR-Cr < 60 mL/min/1.73 m^2^ was excluded from this study. We previously reported that TNFR levels increased with declining eGFR-CysC in patients with type 2 diabetes and normal kidney function, and that the increase in serum TNFRs might result from their increased systemic production, including in the kidney, rather than being a simple reflection of GFR decline^19^. Building on this previous work, the present study demonstrated that the correlation between TNFR levels and eGFR-CysC was observed even in healthy subjects with normal kidney function.

Franceschi et al.^20^ proposed the concept of *inflammaging*, referring to chronic low-grade inflammation that is exacerbated with age and affects the acquired and innate immune systems. The elderly exhibit an inflammaging-like phenotype featuring increases in inflammatory markers such as inflammatory cytokines in the blood and activation of inflammatory signals in the tissues, suggesting the induction of chronic low-level inflammation^21^. In the present study, eGFR-CysC did not change from age 20 to 49, but decreased after age 50. Therefore, age-related elevation in TNFR levels after the age of 50 may simply reflect declining eGFR-CysC, given the finding of multivariate analysis that the relationship between TNFR2 and age disappeared after adjustment for eGFR-CysC. In other words, the increase in inflammatory markers with aging may instead reflect the effects of declining kidney function. Further research is needed to elaborate on the exact mechanisms involved in the increase of TNFRs.

A post hoc analysis of cardiovascular outcome trials involving relatively preserved kidney function in patients with type 2 diabetes treated with a sodium-glucose co-transporter protein-2 (SGLT2) inhibitor, canagliflozin, reported two major findings. Not only were baseline TNFR levels associated with the progression of kidney disease, but also a smaller difference in TNFR levels between baseline and 1 year after SGLT2 inhibitor treatment was associated with better subsequent kidney outcomes^22,23^. Moreover, it has been reported that TNFR levels are associated with histological findings in renal tissues, such as mesangial fractional volume and percentage of endothelial cell fenestration, in early diabetic nephropathy^24^. Therefore, the measurement of TNFR levels may be valuable not only for predicting kidney biopsy findings and future kidney outcomes, but also as a marker of treatment responsiveness, suggesting potential therapeutic applications. Additionally, the association between longitudinal changes in TNFR and the subsequent risk of end stage kidney disease or kidney function decline has been reported in studies such as the VA NEPHRON-D trial involving advanced DKD patients and the AASK trial targeting CKD patients with hypertension^25^. A report has also been published indicating that the Janus kinase inhibitor baricitinib lowers TNFR levels in patients with type 2 diabetes, while another report suggested that the renin-angiotensin-system inhibitor losartan does not have the same effect^26^. There is thus a need for further investigation to determine how TNFR concentrations change with different treatments.

As shown in Supplementary Table 5, the levels of TNFR in the Native American Pima tribe with type 2 diabetes appeared to be higher than those in Japanese patients with type 2 diabetes. This could be explained by the Japanese having lower kidney function but similar levels of albuminuria compared with Pima Native Americans^10,24^. Considering how high circulating TNFR2 level and TNFR2 mRNA level are in fat tissue in obese individuals compared with those in others, it cannot be ruled out that BMI is somewhat involved in the elevated TNFR levels observed in Pima Native Americans with type 2 diabetes^27^. However, in the present study, BMI was not associated with TNFR levels in either healthy subjects or patients with type 2 diabetes, suggesting that this tribe’s TNFR levels are elevated by genetic or other factors.

The impact of various factors on TNFR levels in patients with diabetes makes it difficult to definitively state that these levels independently differ between Japanese and Caucasian populations. However, the TNFR levels in Japanese patients with type 2 diabetes tend to be lower than those in their Caucasian equivalents^28–30^. Further validation is needed to determine whether there are ethnic differences in TNFR levels and what clinical factors influence TNFR levels.

Our study is associated with several limitations that warrant mentioning. We used serum cystatin C-based estimates of eGFR, which are less accurate than direct measurements. Moreover, only Japanese subjects were enrolled in this work. Therefore, the results may not apply to other ethnic groups or DKD patients with kidney function decline. Furthermore, we assembled the healthy subjects from among those who worked at Juntendo University Hospital. Therefore, many of the recruited subjects are likely to have a better understanding of disease than the general healthy population. As such, this control group may have been healthier than the general healthy population.

In conclusion, we revealed that the serum TNFR levels in healthy Japanese subjects are not affected by age or sex, but are related to eGFR even in those with normal kidney function. Further studies are needed to collect a larger number of appropriate subjects from multiple centers and to clarify reference values not only in healthy subjects from the Japanese population but also from other ethnic groups.

### Supplementary Information

Supplementary Information 1.

## Data Availability

The datasets used and analyzed during the current study are available from the corresponding author on reasonable request.
