# Supplementary material for: Circulating TNF receptor levels are associated with estimated glomerular filtration rate even in healthy individuals with normal kidney function
Source: Sci Rep. 2024 Mar 27;14:7245. doi: 10.1038/s41598-024-57265-x (PMC10973483; doi:10.1038/s41598-024-57265-x)
Supplement: Supplementary file 1 — Supplementary Information 1. [file 41598_2024_57265_MOESM1_ESM.pdf]

Supplementary Table 1. Multivariate regression analysis of the factors associated with tumor necrosis factor receptor 1 or tumor necrosis factor receptor 2 in healthy subjects.

TNFR1

Variables	Estimate	SE	t value	P
eGFR-CysC	-0.0019	0.0002	-8.859	<0.0001
eGFR-Cr	-0.00007	0.0003	-0.229	0.82

TNFR2

Variables	Estimate	SE	t value	P
eGFR-CysC	-0.0018	0.0002	-7.782	<0.0001
eGFR-Cr	-0.0001	0.0004	-0.290	0.77
Age	-0.0005	0.0004	-1.047	0.30
Systolic BP	0.0006	0.0003	1.968	0.0498

Abbreviations: BP, blood pressure; Cr, creatinine; CysC, cystatin C; eGFR, estimated glomerular filtration rate; SE, standard error; TNF, tumor necrosis factor; TNFR, TNF receptor.

Supplementary Table 2. Multivariate regression analysis of the factors associated with tumor necrosis factor receptor 1 or tumor necrosis factor recetor 2 in patients with type 2 diabetes.

TNFR1

Variables	Estimate	SE	t value	P
eGFR-CysC	-0.0016	0.0003	-5.69	<0.0001
eGFR-Cr	-0.0006	0.0004	-1.48	0.14
Age	0.0004	0.0007	0.56	0.57
BMI	0.0013	0.0013	1.06	0.29
HbA1c	0.0191	0.0049	3.9	0.0001
Uric acid	0.0175	0.0048	3.52	0.0003
Non-HDL-C	-0.0004	0.0002	-2.41	0.02
UACR	0.0482	0.0086	5.62	<0.0001

TNFR2

Variables	Estimate	SE	t value	P
eGFR-CysC	-0.0017	0.0003	-6.01	<0.0001
eGFR-Cr	-0.0003	0.0004	-0.66	0.51
Age	0.0011	0.0007	1.48	0.14
HbA1c	0.0122	0.00535	2.27	0.02
Uric acid	0.0128	0.0052	2.45	0.01
HDL-C	-0.0023	0.0005	-4.4	<0.0001
Non-HDL-C	-0.0005	0.0002	-2.8	0.006
UACR	0.0345	0.0091	3.78	0.0002

Abbreviations used in this table are the same as in Supplementary Table 1.
Abbreviations: BMI, body mass index; HbA1c, glycated hemoglobin; HDL-C, high-density-lipoprotein cholesterol; UACR, urinary albumin-to-creatinine ratio.

Supplementary Table 3. Spearman's correlation between tumor necrosis factor receptors and clinical parameters in healthy subjects and patients with type 2 diabetes.

Variables	Healthy subjects and diabetes (n = 705)	
	TNFR1	TNFR2
Age	0.37***	0.45***
BMI	0.34***	0.33***
Systolic BP	0.35***	0.39***
Diastolic BP	0.25***	0.30***
HbA1c	0.52***	-0.57***
HDL-C	-0.33***	-0.35***
Non-HDL-C	-0.07	-0.09*
Uric acid	0.11**	0.09*
UACR	0.42**	0.44***
GFR-Cr	-0.10**	-0.10**
GFR-CysC	-0.43***	-0.43***
TNFR2	0.81***	–

*p<0.05, **p<0.01, ***p<0.001

Abbreviations used in this table are the same as in Supplementary Table 1 and Table 2.

Supplementary Table 4. Multivariate stepwise regression analysis of the factors associated with tumor necrosis factor 1 or tumor necrosis factor 2 in healthy subjects and patients with type 2 diabetes.

TNFR1

Variables	Estimate	SE	t value	P
BMI	0.0013	0.0009	1.466	0.14
HbA1c	0.0025	0.0034	7.422	<0.0001
HDL-C	-0.0008	0.00023	-3.520	0.0005
Non-HDL	-0.0005	0.0001	-4.645	<0.0001
Uric acid	0.0094	0.0029	3.210	<0.0001
eGFR-CysC	-0.0019	0.0002	-11.622	<0.0001
eGFR-Cr	-0.0005	0.0002	-2.095	0.04
ACR	0.0441	0.0068	6.444	<0.0001

TNFR2

Variables	Estimate	SE	t value	P
Age	0.0010	0.0004	2.833	0.005
HbA1c	0.0294	0.0037	7.850	<0.0001
HDL-C	-0.0008	0.000247	-3.381	<0.0001
Non-HDL	-0.0006	0.0001	-5.535	<0.0001
Uric acid	0.0104	0.0031	3.379	0.0008
SBP	0.0004	0.0002	1.782	0.08
eGFR-CysC	-0.0019	0.0002	-9.894	<0.0001
ACR	0.0383	0.00752	5.087	<0.0001

Abbreviations used in this table are the same as in Supplementary Table 1 and Table 2.

Supplementary Table 5. TNFR levels in patients with diabetes.

Author	Type of diabetes	n	Trial, study, and/or ethnicity	Age (yrs)	HbA1c (%)	eGFR-Cr or mGFR (mL/min/1.73 m^2)	eGFR-CysC (mL/min/1.73 m^2)	Median UACR or UAER**	TNFR1 (pg/mL)	TNFR2 (pg/mL)	Vendor (ELISA Cat. No.)***	References
Gohda T, et al.	2	292	Japanese	57±11	7.5±1.2	85±19	97±23	18 (7, 59) mg/gCr	1357 (1133, 1646)	2904 (2505, 3391)	R&D Systems (DRT100, DRT200)	Present study
Kamei N, et al. [12]	2	594	Japanese	55±13	7.3±1.2	69 (56, 84)	—	22 (9, 123) mg/gCr	1562 (1263, 2016)	3339 (2717, 4297)	R&D Systems (DRT100, DRT200)	Sci Rep. 8: 15302, 2018.
Murakoshi M, et al. [10]	2	201	Japanese	67±10	7.2±1.0	73±25	—	23 (8, 142) mg/gCr	1412 (1153, 1902)	Not measured	R&D Systems (DRT100)	Front Endocrinol. 13: 849457, 2022.
Saulnier PJ, et al. [28]	2	1135	SURDIAGENE study, French	64±11	7.8±1.5	76±21	—	23 (9, 92) mg/gCr	1818 (1544, 2231)	Not measured	EKF Diagnostics (BIO94TNFR1)	Diabetes Care. 40: 367-374, 2017.
Oshima M, et al. [29]	2	330	ADVANCE and ADVANCE-ON studies	68±7	7.6±1.6	67±16	—	15 (7, 37) mg/gCr	1429 (1150, 1760)	Not measured	R&D Systems (DRT100)	KI Report. 6: 284-295, 2021.
Pavkov ME, et al. [24]	2	83	Pima Indians	46±10	9.2 (7.6, 11.2)	119 (94, 155)*	—	26 (12, 127) mg/gCr	1500 (1205, 1960)	3283 (2670, 4151)	R&D Systems (DRT100, DRT200)	Kidney Int. 89: 226-234, 2016.
Pavkov ME, et al. [30]	2	193	Pima Indians	46 (39, 53)	9.6 (7.7, 11.1)	120 (88, 149)*	—	72 (19, 493) mg/gCr	2833 (2081, 4092)	4835 (3875, 6997)	R&D Systems (DRT100, DRT200)	Kidney Int. 87: 812-819, 2015.
Gohda T, et al. [6]	1	275	First Joslin Kidney Study, Caucasians	31±8	8.9±1.5	—	133±30	56 (37, 101) μg/min**	1345 (1156, 1598)	2161 (1732, 2673)	R&D Systems (DRT100, DRT200)	J Am Soc Nephrol. 23: 516-524, 2012.
Gohda T, et al. [6]	1	353	Second Joslin Kidney Study, Caucasians	39±12	8.4±1.3	—	129±30	41 (24, 79) μg/min**	1382 (1180, 1709)	2230 (1869, 2695)	R&D Systems (DRT100, DRT200)	J Am Soc Nephrol. 23: 516-524, 2012.

Abbreviations used in this table are the same as in Supplementary table 1.

Data are presented as mean ± standard deviation (SD) or median (quartiles). n, number of patients; *mGFR, measured GFR (urinary clearance of nonradioactive iothalamate); **AER (mg/min), albumin excretion rate

***DRT100 (R&D Systems) and BIO94TNFR1 (EKF Diagnostics) are the same ELISA kit, although the vendor is different.

Supplementary Figure 1

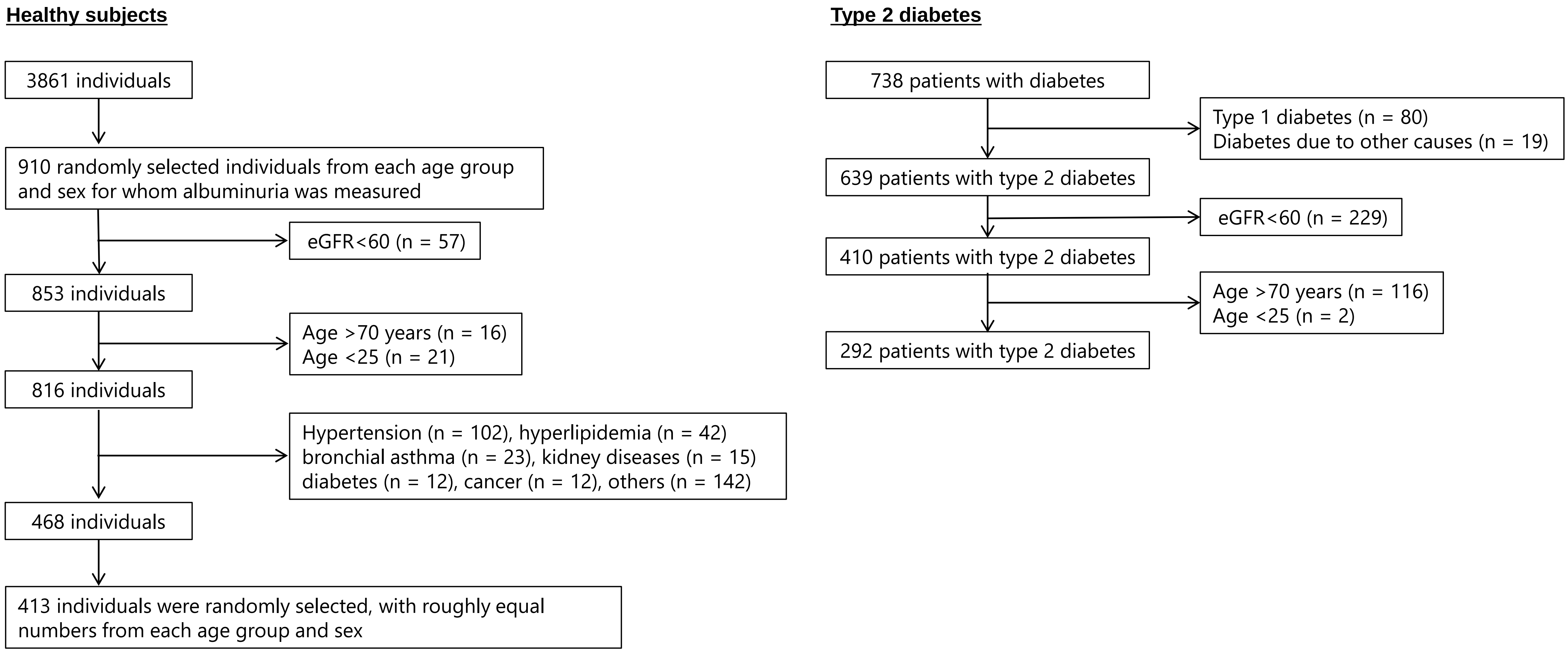

Figure legend

Supplementary Figure 1. Flowchart of inclusion and exclusion of subjects in this study.
